# Venomous Bites, Stings and Poisoning by European Vertebrates as an Overlooked and Emerging Medical Problem: Recognition, Clinical Aspects and Therapeutic Management

**DOI:** 10.3390/life13061228

**Published:** 2023-05-23

**Authors:** Giovanni Paolino, Matteo Riccardo Di Nicola, Ignazio Avella, Santo Raffaele Mercuri

**Affiliations:** 1 Unit of Dermatology and Cosmetology, I.R.C.C.S. San Raffaele Hospital, Via Olgettina 60, 20132 Milan, Italy; paolino.giovanni@hsr.it (G.P.);; 2 Unit of Clinical Dermatology, Università Vita-Salute San Raffaele, Via Olgettina 60, 20132 Milan, Italy; 3 Asociación Herpetológica Española, Apartado de Correos 191, 28911 Leganés, Spain; 4 CIBIO, Centro de Investigação em Biodiversidade e Recursos Genéticos, InBIO Laboratório Associado, Campus de Vairão, Universidade do Porto, 4485-661 Vairão, Portugal; ignazio.avella@cibio.up.pt; 5 Departamento de Biologia, Faculdade de Ciências, Universidade do Porto, 4099-002 Porto, Portugal; 6 BIOPOLIS Program in Genomics, Biodiversity and Land Planning, CIBIO, Campus de Vairão, 4485-661 Vairão, Portugal

**Keywords:** amphibian poison, reptile venom, snakebite, fish sting, venomous mammal, snake venom, stingray, weeverfish, viper

## Abstract

Europe presents a high number of venomous and poisonous animals able to elicit medically relevant symptoms in humans. However, since most of the accidents involving venomous or poisonous animals in Europe are unreported, their incidence and morbidity are severely overlooked. Here we provide an overview of the European vertebrate species of greatest toxicological interest, the clinical manifestations their toxins can cause, and their treatment. We report the clinical symptoms induced by envenomations and poisoning caused by reptiles, fishes, amphibians and mammals in Europe, ranging from mild, local symptoms (e.g., erythema, edema) to systemic and potentially deadly. The present work constitutes a tool for physicians to recognize envenomation/poisoning symptoms caused by the most medically relevant European vertebrates and to decide which approach is the most appropriate to treat them.

## 1. Introduction

Envenomations caused by bites and stings from venomous animals pose a major public health problem in children and adults worldwide [1]. Their impact depends on several factors, such as animal species involved, size and general health condition of the envenomated person, and accessibility to appropriate health care [1,2,3]. A number of studies have suggested that the number of animal-related accidents is likely to rise in the future due to climate change [1,2,4,5,6,7]. Indeed, with climate change, several animal species could change their ecology and distributional ranges, potentially leading to an increase in the coming years in the number of encounters between humans and venomous animals, causing an increase in morbidity and mortality [1,2].

Considering that reporting bites and stings of venomous/poisonous animals is not mandatory in the European health system, information concerning most of them is often unavailable [3]. In light of this, and of the relatively high number of medically relevant European animal species, incidence, morbidity and mortality caused by venomous and poisonous vertebrates in this continent is likely underestimated. Several European invertebrate species, for example, are known to cause relevant medical manifestations in humans thanks to their venomous stings and bites (e.g., jellyfish [8,9,10,11], scorpions [12,13] and spiders [14,15,16,17]). Similarly, the toxins produced by a number of European vertebrate taxa are able to elicit symptoms ranging from minimal and local (e.g., pain, swelling), to systemic (e.g., cardiovascular, hematological, neurological, gastrointestinal) and sometimes deadly [1].

Unfortunately, the international medical scientific literature greatly neglects this topic, and the majority of physicians are thus generally not trained in the identification of noxious animal species and in treating the intoxication symptoms they can cause. Hence, non-trained physicians usually need to consult Poison Control Centers (PCCs) before appropriately treating envenomated/poisoned patients, thus not guaranteeing immediate treatment. In the worst-case scenario, they could even adopt therapeutic methods not appropriate to the case, potentially even causing greater harm to the patients than the toxins themselves [3].

In this scenario, considering the need for an intervention aimed at educating physicians in the management of clinical symptoms caused by European venomous and poisonous vertebrates, we provide here an overview of the main features of the European vertebrate taxa most commonly causing medically relevant symptoms in humans, the local and systemic manifestations of the envenomation they can cause, and their treatments.

## 2. Materials and Methods

**Figure 1:**
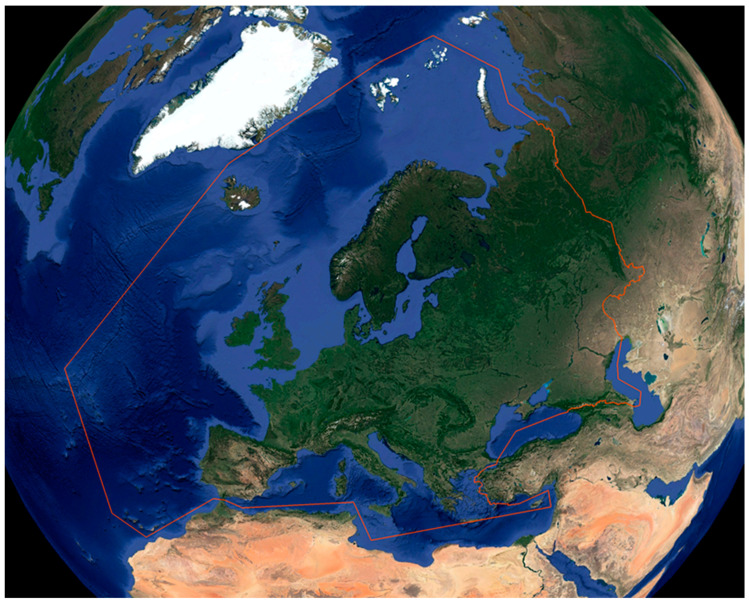
European area considered. Map credit: Google Earth (modified).

Vertebrates comprise all animal taxa within the subphylum Vertebrata (chordates with backbones), including Agnatha (hagfish and lampreys) and Gnathostomata (cartilaginous fish, bony fish, amphibians, reptiles, birds and mammals). In the present work, we will focus on the most common venomous and poisonous vertebrate taxa that cause medically relevant reactions in Europe. We only considered vertebrates that, through bites, stings or contact, may cause local and/or systemic manifestations due to the effect of their toxins. According to Speybroeck et al. [18], the European area considered in this work includes all territories of the European mainland, Macaronesia (except Cape Verde), the Balearic Islands, all Greek and Italian islands, Malta and Cyprus (Figure 1).

Publications consulted for the current study were gathered using the PubMed web search engine (https://pubmed.ncbi.nlm.nih.gov/, accessed on 28th March 2023) and the Cochrane Central Register of Controlled Trials (CENTRAL) web search engine from EMBASE (available at https://www.cochranelibrary.com/central, accessed on 28 March 2023).

The following strings were searched on PubMed: (“snake bites”, “weever sting”, “stargazer fish sting”, “dog fish sting”, “stingray sting”, “Malpolon bite”, “Bufo poisoning”, “Salamandra poisoning”, “Bombina poisoning” [MeSH Terms] OR (“vertebrate” [All Fields] AND “bites” [All Fields]) OR “vertebrate bites” [All Fields] OR “vertebrate stings” [All Fields] OR. 

(“bites” [All Fields] AND “stings” [All Fields])OR (“vertebrate” AND “poisoning” AND “envenoming”) AND (“viper” OR “scorpion” OR “squalus” OR “dasyatidae” OR “uranoscopidae”). For the search performed using CENTRAL, the following terms were searched: “Europe”, “venom”, “poison”, “vertebrate”, “Salamandra”, “Bufo”, “toad”, “dogfish”, “weever”, “stargazer fish”. 

Given that cases of venomous bites, stings and poisoning are often unreported in Europe, we found a relatively low number of reports from the literature. Therefore, we designed and structured our work as a more conceptual review, aiming to provide a general overview of envenomation/poisoning caused by vertebrates in Europe and their treatment, and raise awareness of this often neglected medical problem. Table S1 summarizes the main characteristics of distribution, clinical signs, clinical management and possible availability of antivenoms for each taxa considered.

## 3. Results

From the existing literature consulted, cartilaginous fish (class: Chondrichthyes), bony fish (classes: Actinopterygii and Sarcopterygii), amphibians (class: Amphibia), reptiles (class: Reptilia) and mammals (class: Mammalia) appeared to be the vertebrate classes of highest toxicological interest in Europe. For each class, we here report the main taxa involved in medically relevant accidents attributable to the injection/absorption of toxins, a brief zoological framework, the main clinical aspects of envenomation/poisoning caused by bites/stings/contact, pathogenesis and the corresponding treatment. 

### 3.1. Reptiles

The only venomous reptiles existing in Europe able to cause medically significant symptoms in humans belong to the suborder Serpentes (i.e., snakes). Current, conservative estimates suggest that snakebites alone kill between 81,000 and 138,000 people and cause long-lasting disabilities in about 421,000–1,000,000 people worldwide every year [19], a burden of death and disability comparable to that of prostate and cervical cancer [3,19,20]. Front-fanged snakes (i.e., families Viperidae, Elapidae and Atractaspididae) are generally considered to be the most dangerous venomous snakes (see [21]), and are accountable for the vast majority of medically relevant ophidic accidents [22,23,24]. Although the only front-fanged snakes present in Europe are viperids (family: Viperidae), the continent also hosts some rear-fanged venomous snakes [25,26,27,28,29], generally considered medically neglectable but still able to cause clinically relevant symptoms with their bites [30,31,32].

#### 3.1.1. Genus *Vipera*

**Figure 2:**
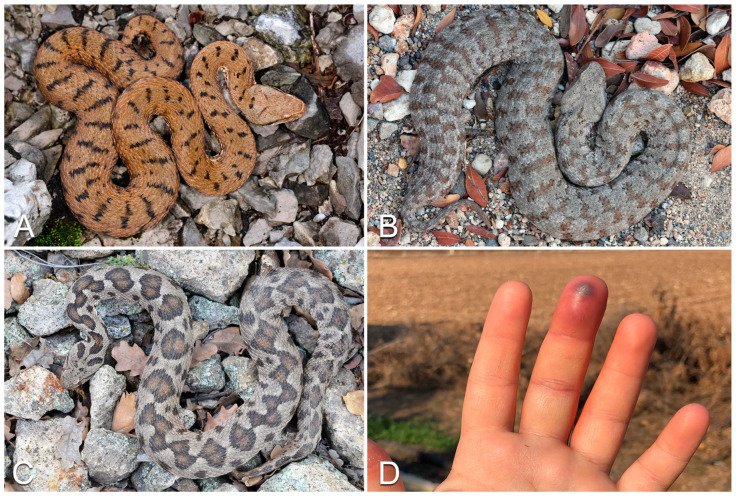
Examples of European vipers: (**A**) *Vipera aspis*; (**B**) *Macrovipera schweizeri*; (**C**) *Montivipera xanthina*. (**D**) Clinical signs following *Vipera aspis* bite: erythema, ecchymosis and necrosis in the finger. Photo credit: Matteo R. Di Nicola (**A**–**C**); Sebastian Colnaghi (**D**).

The genus *Vipera* (Figure 2A) belongs to the “true vipers” (subfamily: Viperinae) and includes more than 20 species distributed across the Old World. In Europe, *Vipera* occurs in virtually every country from Portugal to Russia, including Mediterranean islands (i.e., Sicily, Elba and Montecristo) and the United Kingdom [25,33], but is not present in some large islands such as the Balearics, Corsica, Crete, Malta and Sardinia [26,27,28,34]. For the detailed distribution of each European species, see Di Nicola et al., (2022) [28] and Speybroeck et al., (2016) [26]. Members of this genus are front-fanged, medium-sized (from 40 cm to roughly 100 cm) snakes with a typically stocky body, sub-triangular or sub-oval head distinct from the neck, eyes with vertical elliptical pupil, dorsal scales always keeled and a relatively short tail [3,26,28,35]. Being ambush predators, members of the genus *Vipera* are often difficult to spot in the wild because of their highly effective camouflage and often end up biting and envenomating people accidentally stepping on them [19,36,37,38,39].

#### 3.1.2. Clinical Features of the Bites

In Europe, the *Vipera* species most frequently involved in snakebite accidents is *Vipera berus* [19]. The main dermatological manifestations of envenomations caused by snakes of this genus are characterized by the typical presence of fang marks (1–2 distinct holes in the bite site, caused by the fangs) and associated with extended erythema, edema and pain; cutaneous necrosis, hives, purpura, petechiae and acute compartment syndrome may appear in later stages (Figure 2D). After local manifestations, systemic symptoms may also occur, including fatigue, pain, fever, direct anaphylactoid reaction, anxiety, cranial nerve neurotoxicity, dysesthesia/paraesthesia, vomiting, arrhythmia, cardiac ischemia, abdominal pain, diarrhea, dyspnea, proteinuria, hematuria, secondary infections and disseminated intravascular coagulation. “Dry bites” (i.e., bites without the injection of venom) may occur, creating alarmism in the patient and physicians, although without clinical consequences [20].

#### 3.1.3. Pathogenesis

The pathogenesis of *Vipera* snakebites varies according to the degree of variation in venom composition: indeed, snake venom composition varies at different levels [40,41,42]. Generally, viperid venoms mainly induce hemotoxic and cytotoxic symptoms, and sometimes also present neurotoxic effects. The major *Vipera* toxins are phospholipases A2 (capable to induce a plethora of symptomps, such hemolysis, neurotoxicity, myotoxicity, cardiotoxicity, cytotoxicity, anticoagulation, convulsions, hypotension and inflammation); snake venom serine proteinases (thrombin-like enzymes, activators of prothrombin, factor V and factor X) and snake venom metalloproteinases (causing local and systemic hemorrhages, although some classes of snake venom metalloproteinases may also induce pro-coagulant and pro-inflammatory activities); snake C-type lectin-like proteins (anticoagulant- and platelet-modulating activities); and disintegrins (cell adhesion, migration, apoptosis, platelet aggregation and angiogenesis) [3,20,43,44].

#### 3.1.4. Clinical Management

After a *Vipera* snakebite, the following laboratory investigations should be always performed: hemocoagulation, blood count, urinalysis, hepatic tests, renal function, electrolytes, LDH and CPK. All these laboratory investigations should be performed at Time 0 and every 6 h for 24 h, together with digital oximetry and electrocardiogram (ECG). The antivenom (Table S1) is prescribed in case of envenomation ≥G1 [3,20]. The cutaneous area affected can be cleaned with hydrogen peroxide, potassium permanganate or even plain water (snake venom is water soluble). The use of tourniquets, cutting, sucking or scarifying the wound and the application of chemicals or electric shock are not advised. Antibiotic treatment is justified only in case of ascertained superinfections. Tetanus immunization status should always be evaluated and immunization provided as needed. Benzodiazepines can help calm the patient by reducing anxiety. It is very important to perform an accurate clinical evaluation of vesicles and blisters, since sometimes their extension can be symptomatic of an underlying necrosis.

#### 3.1.5. Genus *Macrovipera*

All members of the genus *Macrovipera* are robust, thickset vipers (Figure 2B). Species of this genus present a large head, clearly distinct from the neck, and a characteristically rounded snout. Individuals of this genus in Europe are found only in the Milos archipelago (Greece) and Cyprus, although they are mainly distributed in Wstern Asia [26,28,39]. To date, several cases of envenomation caused by *Macrovipera* bites have been reported [45,46,47,48].

#### 3.1.6. Clinical Features of the Bites

A few minutes to a few hours after a bite, erythema and edema arise, accompanied by bruising with lymphangitic lines and painful regional lymphadenopathy spreading rapidly to encompass the envenomed limb and part of the trunk [45]. The clinical–instrumental investigations required to monitor the victim are the same as those for the genus *Vipera*. Coagulopathy and thrombocytopenia may lead to extensive ecchymosis, subconjunctival and retinal hemorrhages, hemoptysis, and melena, resulting in severe anemia. The estimated fatality induced by *Macrovipera* bites is high, reaching 50% mortality rates [48], although this is estimate is based on a small sample size and thus likely misleading.

#### 3.1.7. Pathogenesis

*Macrovipera* venom is mainly characterized by hemotoxic and cytotoxic effects induced by high amounts of snake venom metalloproteinases (SVMPs), snake venom serine proteinases (SVSPs), phospholipases A2 (PLA2s), L-Amino acid oxidases (LAAOs) and C-type lectins (CTLs), together with many other toxin families (e.g., disintegrins, cysteine-rich secretory proteins) [39,43,49,50,51,52,53].

#### 3.1.8. Clinical Management

The bites of *Macrovipera* spp. require immediate and timely hospital treatment. The clinical–instrumental monitoring of the victim is the same as reported forthe genus *Vipera*. Given the potential severity of bites inflicted by this genus, the chances of having to administer an antivenom are high. Hemorrhagic blisters may arise, likely followed by local necrosis. Falling blood pressure and tachycardia, possible symptoms of *Macrovipera* bites, can develop into shock. Envenoming caused by *Macrovipera* spp. can often require a multidisciplinary approach (dermatological, surgical, hematological and above all cardiological), given the possibility of different systemic manifestations.

#### 3.1.9. Genus *Montivipera*

Members of the genus *Montivipera* are long (maximum total length up to 120–130 cm), robust vipers [26,54], morphologically quite similar to snakes of the genus *Macrovipera* (see Di Nicola et al., (2022) [28] for detailed morphological differences between the two genera; Figure 2C). Vipers of the genus *Montivipera* are mostly located in the Middle East, while in Europe they inhabit only few areas in northeastern Greece and some Aegean islands [25,26,28]. The genus *Montivipera* represents a higher medical threat in the eastern part of its range rather than in the European continent [55,56,57,58].

#### 3.1.10. Clinical Features of the Bites

The bite of *Montivipera* species can induce severe cutaneous and systemic complications. Erythema and edema arise in the bite site, followed by rapid spreading, extensive swelling, local ecchymosis, blistering and necrosis. Systemic symptoms, when present, include diffuse myalgias, anemia, coagulopathies and neurological and urological symptoms [55,56,57,58].

#### 3.1.11. Pathogenesis

To date, little is known about the toxin components of *Montivipera* venoms. Based on the information currently available, it presents a typical “viperid-like” composition, being mainly characterized by bradykinin-potentiating peptides, C-natriuretic peptide, disintegrins, Zn2+-metalloproteinase, serine proteinase, L-amino acid oxidase; phospholipase A2, cysteine-rich secretory proteins and Kunitz-type inhibitor [43,56].

#### 3.1.12. Clinical Management 

The bitten area should be cleaned with plain water or antiseptic agent (hydrogen peroxide or potassium permanganate). Clinical and instrumental investigations are the same for *Vipera* spp. and *Montivipera* spp. Antivenom should be prescribed in case of local worsening of the bitten area and in case of the onset of systemic symptom. Indeed, despite the size of *Montivipera* spp., envenomings attributable to members of this genus are rarely lethal unless secondary complications involving vital organs arise or antivenom therapy is delayed or unavailable [56].

#### 3.1.13. Genus *Malpolon*

**Figure 3:**
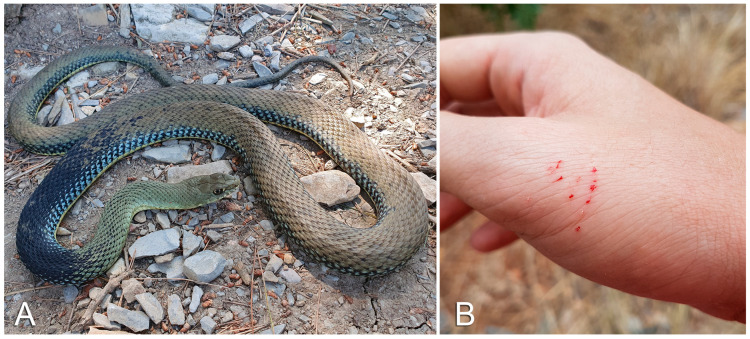
(**A**) *Malpolon monspessulanus*. (**B**) Small, linear, multiple cutaneous erosions with bloody discharge after a superficial *Malpolon monspessulanus* bite. Photo credit: Matteo R. Di Nicola.

Members of the genus *Malpolon* are long, thickset, rear-fanged snakes (Figure 3A). Their European distribution encompasses the Balkans, Greece, Portugal, Spain, southern France, Mediterranean northwestern Italy and some Mediterranean islands [25,26,28,29]. Although generally considered able to elicit only mild, local envenomation symptoms, snakes of this genus have been reported to sometimes cause more serious disturbances [30,31].

#### 3.1.14. Clinical Features of the Bites 

In order to inject venom through their bite, members of the genus *Malpolon* generally need to hold onto the bitten part and chew it for some time (i.e., prolonged bite). A quick, shallow bite usually results in only superficial grazes (Figure 3B). In case of a prolonged bite, local symptoms involving the bitten area are erythema, edema and pain. Systemic symptoms, including neurotoxic effects (e.g., oculomotor paralysis), have sometimes been reported following *Malpolon* envenomations [30,31,59].

#### 3.1.15. Pathogenesis 

*Malpolon* spp. venom is partially unknown. To date, snake venom metalloproteinases, peptidase M1 and cysteine-rich secretory proteins are thought to be its most prominent components [30,31,59].

#### 3.1.16. Clinical Management 

*Malpolon* venom is generally considered of limited medical relevance, and no antivenom is currently available against it. Treatment of *Malpolon* spp. bites is exclusively symptomatic.

#### 3.1.17. Other Snakes

The presence of small venom glands (typically referred to as Duvernoy’s glands) is known in several European rear-fanged colubrids [60,61,62,63,64,65,66]. Among these, members of the genera *Macroprotodon* (distribution in Europe: Iberian Peninsula, Balearic Islands and Lampedusa) and *Telescopus* (distribution in Europe: Balkan peninsula, Greece, Cyprus, Malta, northeastern Italy) [26,28] are only rarely reported to cause mild, local symptoms (e.g., swelling, erythema, pain) following their bites, and will therefore not be discussed in detail. In cases of envenomation by snakes of these genera, only topical treatment (e.g., washing the area with plain water, application of antibiotics in case of infection) is recommended.

### 3.2. Fish

Currently, about 2.000 fish species are recognized as venomous [64], although this number is probably underestimated [67]. In this chapter we will only deal with the species most frequently inflicting venomous stings of medical relevance in Europe. 

#### 3.2.1. Trachinidae (Weever Fish)

**Figure 4:**
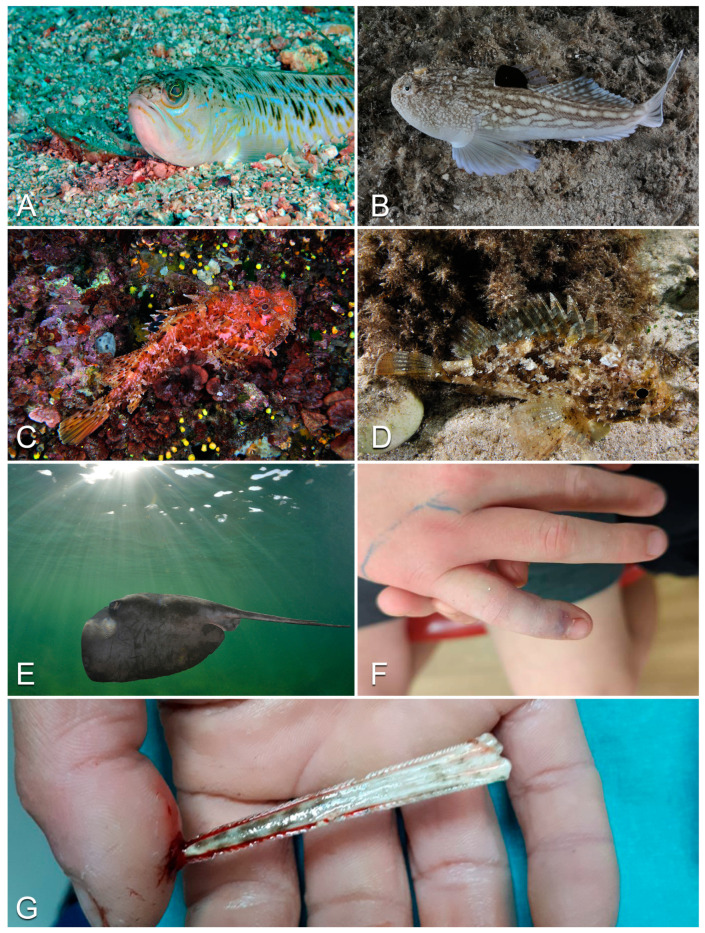
Examples of European venomous fish: (**A**) *Trachinus draco*; (**B**) *Uranoscopus scaber*; (**C**) *Scorpaena scrofa*; (**D**) *Scorpaena porcus*; (**E**) *Pteroplatytrygon violacea*; (**F**) envenomation caused by *Trachinus draco* in a 10-year-old boy. Note the presence of the small puncture in a finger, characterized by ecchymosis and by an erythematous and swelling surrounding skin; (**G**) stingray harpoon piercing a victim’s finger through the bone. Photo credit: Marco Colombo (**A**–**E**); Verity Freeman (**F**); Mehmet Sait Akar (**G**).

Fishes of the family Trachinidae are long (up to 50 cm), mainly brown in color, and widespread across the muddy bottoms of the Mediterranean and the European Atlantic waters, from Scotland to the Canary Islands [68]. Members of this family present characteristic spines on the opercula and on the first dorsal fin, through which they can inject venom into the body of their attackers/predators (Figure 4A) [69]. A recent work indicates almost 40 patients stung by the weever in Europe [70], suggesting that Trachinidae is the fish family causing the highest number of envenomations [71,72,73,74,75,76,77,78,79,80,81,82,83,84,85,86,87,88,89,90].

#### 3.2.2. Clinical Features of the Stings

The pain caused by weever fish stings is usually excruciating, typically peaking 30–50 min after the sting and often persisting for days. It is first localized to the injection site (often hands and soles of the feet of bathers), but later spreads to other parts of the body. The affected area first becomes erythematous, then edematous (Figure 4F), and necrosis can ultimately occur. Fever, arthralgia, cardiac arrhythmias and tonic–clonic seizures can also arise [71,72,73,74,75,76,77,78,79,80,81,82,83,84,85,86,87,88,89,90].

#### 3.2.3. Pathogenesis

The most abundant toxins are dracotoxin and trachinin, with the first inducing destruction of erythrocytes and necrosis, and the second showing neurotoxic activity [91,92,93]. Other molecules reported in the venomous cocktail from fish of the family Trachinidae are phospahatase, proteinase, serotonin and histamine, which cause nociception and inflammation [92].

#### 3.2.4. Clinical Management

Given that cases where the stung subject faints while in the water are frequent, the patient should be helped with reaching the shore as soon as possible. First of all, it is important to check for the presence of barbs and/or dirt in the sting site and eventually remove them. The area should then be disinfected and immersed in hot water (maximum 40 °C), as high temperature could hamper the effects of weever fish’ thermolabile venom. It is recommended that the affected limb be kept raised. Antibiotic treatment is justified only in case of confirmed superinfections. Tetanus immunization status should always be evaluated and immunization provided as needed. Anti-inflammatory (steroidal or non-steroidal) treatment can be provided to reduce pain, erythema and swelling [70].

#### 3.2.5. Uranoscopidae (Stargazer Fish)

Inhabiting the sandy bottoms of Atlantic, Pacific and Indian Oceans, members of this family also inhabit the Mediterranean and the Red and Black Seas [94,95]. Stargazer fish are distinguishable for having the eyes located on top of their heads and for possessing two venomous spines, one per gill operculum (Figure 4B) [68,68,69,70,71,72,73,74,75,76,77,78,79,80,81,82,83,84,85,86,87,88,89,90,91,92,93,94,96,97,98]. Despite the presence of several case reports in the web [99], it should be noted that the actual presence of venom in stargazer fish spines is sometimes still questioned (see [100]) and that reports concerning envenomations caused by members of the family Uranoscopidae in the official literature appear to be lacking.

#### 3.2.6. Clinical Features of the Stings

Stargazer stings cause acute pain, erythema and edema in the affected area [100]. The sting can also induce shivering, sweating, dizziness, arthralgia, shortness of breath, arrhythmia, convulsions and loss of consciousness, and potentially lead to death [101].

#### 3.2.7. Pathogenesis 

To date, no reliable information is available about the composition of the venom produced by members of this family. 

#### 3.2.8. Clinical Management

The venom of stargazer fish is thought to be thermolabile (see [102]). In this case, the same treatment applied for weever fish stings should be applied for stargazer fish envenomations. Steroids and anti-inflammatory treatment can be given to reduce inflammation. Antibiotics are to be prescribed only in case of superinfection. Tetanus immunization status should always be evaluated, and immunization provided as needed.

#### 3.2.9. *Squalus acanthias* (Spiny Dogfish)

A small-sized shark, the spiny dogfish possesses two dorsal spines, one in front of each dorsal fin, linked to venom-secreting vacuolated cells [93]. Although stings inflicted by *S. acanthias* are known to be painful, to our knowledge no information about this species’ venom is currently available [103], and envenomation cases are more commonly reported from non-European countries [104].

#### 3.2.10. Clinical Features of the Stings 

Symptoms caused by *Squalus* sp. stings include erythema, edema and pain. Although rare, fatal envenomation cases have also been reported [105,106].

#### 3.2.11. Pathogenesis

We could not retrieve any reliable information about the composition of the venom produced by species of the genus *Squalus*.

#### 3.2.12. Clinical Management 

In the absence of specific protocols to treat *Squalus* sp. envenomations, these should be managed symptomatically. The affected area should be treated and disinfected with povidone iodine or sodium chloride. It is advisable to keep the affected limb elevated in order to reduce edema. Ulcers potentially arising at the puncture site should be treated topically and medicated daily.

#### 3.2.13. Scorpaenidae (Scorpionfish)

Scorpionfish (genus *Scorpaena*) are ambush predators characterized by a stocky body, large eyes and mouth, and cryptic coloration (Figure 4C, D). Members of the family Scorpaenidae are distributed across the Indian, Pacific and Atlantic Oceans. In the latter, they are found from the British Isles to the Azores and the Canary Islands, in the Mediterranean Sea and in the Black Sea [107]. Scorpionfish typically live on rocks and coral in reefs, bays and lagoons, where they wait motionless for prey [108]. The scorpionfish’s venom apparatus consists of dorsal, pelvic and anal fin spines with elongated venom glands in their anterior portion [108,109]. When mechanical pressure is applied to the spine, the integumentary sheath covering it retreats, allowing the venom to flow into the stung area [110]. 

#### 3.2.14. Clinical Features of the Stings 

Usually, the area stung by fish of the genus *Scorpaena* is characterized by intense, immediate pain, and quickly becomes cyanotic, erythematous and edematous. After a few minutes, systemic symptoms may arise, such as dyspnea, nausea, vomiting and arrhythmias [111,112]. Cases of neuritis and paralysis are reported, as well as secondary over-infections and tetanus [111,112]. Stings by non-venomous spines can also induce infections if a high percentage of bacteria is present on them.

#### 3.2.15. Pathogenesis 

While the composition of the venoms of several scorpenid species is unknown, the venom of *Scorpaena plumieri* has been thoroughly analyzed. The characterization of the composition of this species’ venom can be considered the first step in understanding the mechanisms of action of other Scorpaenidae fish venoms. The venom extract of *S. plumieri* (referred to as SpV) contains several bioactive proteins, such as gelatinolytic proteinases (Sp-GPs), lectins and cytolytictoxins (Sp-Ctxs) [113,114]. Considerable evidence supports the role of Sp-Ctxs as the main culprits for the cardiovascular, inflammatory and cytolytic effects of *S. plumieri* venoms [115,116], while Sp-Gps are accountable for its inflammatory and edema-inducing effects [117,118], and lectins possess pro-inflammatory activity, induce hemocyte agglutination and disrupt the interaction between cell and extracellular matrix [119]. 

#### 3.2.16. Clinical Management 

Envenomation can be caused by the spines of both live and dead specimens. Scorpionfish toxins are thermolabile [120], so hot water immersion (about 45 °C) of the affected limb for 30–90 min can prove effective [112]. In specific cases, an electrocardiogram can be recommended to examine the electrical activity of the heart and its rhythm. Physiological solution and systemic treatments will be administered to relieve symptoms. Should these prove ineffective, analgesic drugs and steroids will be used. Some authors have found the injection of emetin hydrochloride, potassium permanganate and Congo red at the sting site useful ([121]). Tetanus prophylaxis, systemic and/or local antibiotic therapy should be considered based on the patient’s status. Puncture-induced anaphylactic shock can arise even without prior sensitization, and anti-anaphylaxis measures should thus be considered.

#### 3.2.17. Dasyatidae (Stingrays)

Members of this family occur worldwide in tropical to temperate marine waters, with several species being present in the Atlantic Ocean and the Mediterranean Sea [122,123,124,125]. Stingrays typically present a characteristic flat, diamond-shaped body, eyes perched on top of it and a whip-like tail with upper and lower fin folds (Figure 4E) [97]. On the tail, one or more barbed, harpoon-shaped stingers are covered by an epithelium filled with several venom-secreting glandular cells. When mechanically compressed during penetration, these are unroofed and liberate their toxic content into the tissues of the stung victim [97,126]. Stingrays use their venomous sting to defend themselves against predators and, although generally docile, they commonly cause envenomations in humans, mainly divers and fishermen [127].

#### 3.2.18. Clinical Features of the Stings 

Given that most accidents happen when the subject inadvertently steps on the stingray, most of them involve the lower limb region [127,128]. Damage to the stung subject can occur both by envenoming and by direct damage to tissues and/or bones caused by the stinger (Figure 4G) [127,128,129]. Venom effects typically result in erythema, edema and intense pain at the puncture site [127,128,129]. Systemic symptoms can also occur and can include nausea, vomiting, diarrhea, muscle cramps, dyspnea, cardiac dysrhythmias, hypotension, seizures and convulsions. In the most severe cases, death can occur [127,128].

#### 3.2.19. Pathogenesis 

A variety of proteins, enzymes and serotonergic and cholinergic substances have been identified as components of Dasyatidae venoms. Specifically, proteomic and transcriptomic analyses carried out for the species *Neotrygon kuhlii* identified several protein types in its venom and venom barb tissue, including galectin, cystatin and peroxiredoxin-6 [130]. These proteins are thought to possess apoptotic and pro-inflammatory activity [131], inhibit the defensive enzymes of the envenomated organism [132] and express toxic PLA2 activity [133], respectively. A recent study by Kirchhoff et al. [134] focusing on three stingray species belonging to the family Dasyatidae (*Dasyatis pastinaca*, *Himantura leoparda*, *Pteroplatytrygon violacea*) and two species of the family Potamotrygonidae (*Potamotrygon leopoldi*, *Potamotrygon motoro*) reported an abundance of translationally controlled tumor protein and hyaluronidase, and serine proteinase, metalloproteinase and PLA2, concordant with the inflammatory, tissue- and hemostasis-disrupting activity typically reported for stingray venoms [126,127,130].

#### 3.2.20. Clinical Management

Stingray venom is thermolabile [135]; therefore, immersion of the affected area in hot (43–46 °C) water can reduce the pain and the severity of the envenomation [127,136]. The wound should be evaluated using standard procedures. Surgical removal of foreign bodies (e.g., stinger barbs) might be needed in order to reduce the risk of infections and necrosis [136,137]. Tetanus status should be evaluated, and tetanus immunizations updated if necessary. In the case of thoracoabdominal wounds and/or systemic symptoms, the patient should be immediately referred to care facilities staffed for imaging technologies, critical care management and cardiovascular surgery [127]. Antibiotic prophylaxis should be considered for deep stingray wounds [138].

### 3.3. Amphibians

Amphibian skin comprises numerous secretory glands producing several different toxins used for defensive purposes against predators and external pathogens (e.g., bacteria, fungi) [139,140,141,142,143,144]. 

**Figure 5:**
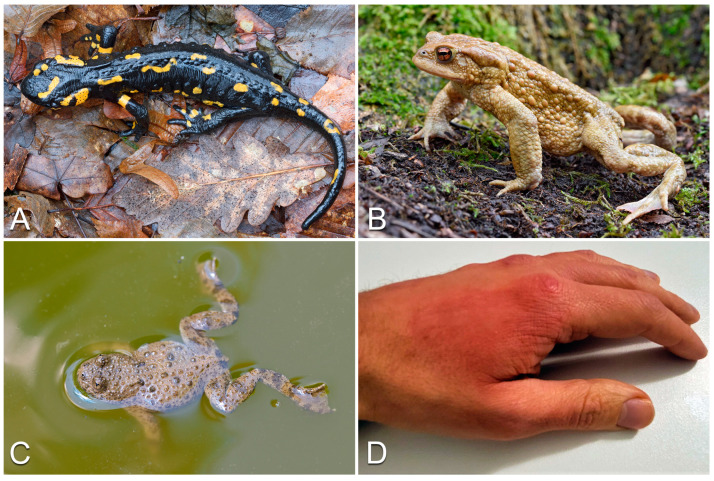
Examples of European poisonous amphibians: (**A**) *Salamandra salamandra*; (**B**) *Bufo bufo*; (**C**) *Bombina variegata*. (**D**) Erythema associated with itch after contact with a *Bombina variegata*. Photo credit: Matteo R. Di Nicola (**A**–**C**); Giovanni Paolino (**D**).

Although all European amphibians are able to secrete different toxins from their skin, only few genera can cause relevant toxic effects in humans, such as *Salamandra* (fire and Alpine/Lanza’s salamanders; mainly European distribution: most of the continent, except the British Isles, much of northern and eastern Europe and the major Mediterranean isles; Figure 5A), *Bufo*/*Bufotes* (common and green toads; mainly European distribution: almost all of the continent, except Ireland and parts of Scandinavia; Figure 5B), *Bombina* (yellow-bellied toads; mainly European distribution: large parts of central, eastern and southeastern Europe; Figure 5C) and *Pleurodeles* (ribbed newts; mainly European distribution: Iberian Peninsula, except the northern mountain areas) [26]. See [145,146,147,148,149,150,151,152] for an overview of the toxins of these taxa. Not having a real toxin-injecting apparatus, amphibians are typically considered poisonous rather than venomous. Nonetheless, newts of the genus *Pleurodeles*, present in North Africa and Iberian Peninsula [153], possess the ability to push their ribs out of their skin while secreting poison. The ribs coated in poison constitute an effective stinging mechanism, injecting toxins into the body of predators/attackers through the puncture wounds they create [154,155]. In light of this, newts of the genus *Pleurodeles* could thus be considered both poisonous and venomous. 

#### 3.3.1. Clinical Features of Toxins 

Poisoning caused by European amphibians generally leads to very mild symptoms, typically not going further than contact rashes. Indeed, after physical contact with the skin of amphibians, erythema and itching might occur with typical aspects of contact dermatitis ([141,156,157]; Figure 5D). In cases of amphibian toxin inhalation, however, respiratory symptoms may also arise, generally consisting of itching in the nose, sneezing, and bronchoconstriction in allergic and atopic subjects (e.g., [156,158]). In cases where the toxins penetrate the skin of the patient, systemic, medically relevant symptoms (e.g., cardiac arrhythmias, hypertension, respiratory insufficiency, blockage of neuromuscular transmission, blockage of the diaphragm muscle, dyspnea, hypotension) might arise (see [147,159,160]).

#### 3.3.2. Pathogenesis

The main known amphibian toxins are bufotaline, bufotenine, bufaline, 5-methoxy-*N*,*N*-dimethyltryptamine, epinephrine, norepinephrine, serotonin, samandarine, tetrodotoxins, bradichynine and defensines [141,161]. In terms of pharmacological effects, these toxins can elicit cardiotoxic, hemotoxic, neurotoxic, myotoxic, hypotensive, hypertensive and anesthetic effects of different severity, depending on the taxon [141,161,162].

#### 3.3.3. Clinical Management 

After contact with amphibian skin, it is good practice to immediately wash the interested area with fresh and running water. It is important not to touch the mucosa (i.e., nose, eyes and mouth) in order to avoid the onset of more important systemic symptoms due to the contact of toxins with the mucous membranes [163,164]. Topical steroids may be useful to reduce erythema and itching. Systemic antihistamines and systemic steroids are useful in case of allergic symptomatology.

### 3.4. Mammals

All the venomous mammals of Europe belong to the order Eulipotyphla, formerly known as Insectivora, and are mostly members of the family Soricidae (i.e., shrews). The venom delivery system of all species of this order consists of large granular submaxillary glands producing toxic saliva, which flows into the body of the prey/attacker through the wounds their sharp teeth can cause [165,166].

**Figure 6:**
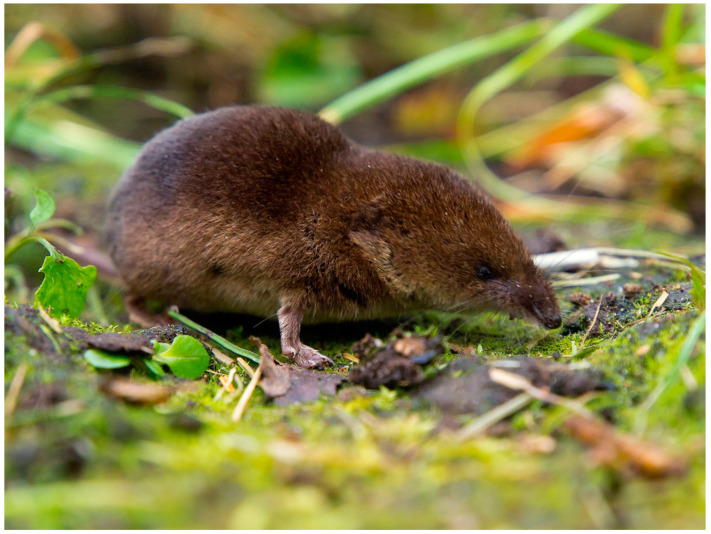
Example of European venomous mammal: common shrew (*Sorex araneus*). Photo credit: Saxifraga-Rudmer Zwerver.

Among the European Eulipotyphla, only the three shrew species *Sorex araneus* (Figure 6), *Neomys anomalus* and *Neomys fodiens* have actually been confirmed to be venomous [167,168,169]. The species *Neomys teres* is considered highly likely to also be venomous, given its ecological and morphological similarities to *N. fodiens* [170], but studies aimed at verifying this are currently lacking. Observations seem to suggest that the Canarian shrew *Crocidura canariensis* and the European mole *Talpa europaea* could also be venomous. Specifically, *C. canariensis* has been shown to be able to induce paralysis in lizards through its bite [171], while *T. europaea* (family Talpidae) presents large, granular maxillary glands and caches paralyzed invertebrate prey in burrows for later consumption [166]. Nonetheless, the venomousness of these two species has yet to be tested.

#### 3.4.1. Clinical Features 

While European shrew bites are not considered a relevant threat to human health, mainly causing discomfort at the bite site [172], they have been shown to potentially cause systemic effects in different target animals. For example, *N. anomalus* and *N. fodiens* salivary extracts injected intracerebrally and intravenously into mice, voles and rabbits have been shown to affect the nervous system, causing paralysis of the hind limbs, spasms, convulsions, general depression, respiratory failure and drop in blood pressure [173,174]. Analyses of in vitro toxicity of *N. fodiens* venom on frogs and beetles confirmed its paralytic activity and cardioinhibitory effects [167]. Additionally, a recent study found the venoms of *N. fodiens* and *S. araneus* to possess hemolytic activity [168].

#### 3.4.2. Pathogenesis 

The analysis of *N. fodiens* venom has led to the recognition of components such as phospholipase A2 (PLA2), disintegrin, metalloproteinase domain-containing protein (ADAM), lysozyme C and hyaluronidase [167,168]. Specifically, PLA2 can determine the insurgence of different effects, including paralysis, cardio- and neurotoxicity [175], while ADAM affect cell adhesion and proteolysis [176]. Lysozyme C is involved in antimicrobial defense [177], and hyaluronidase likely facilitates the spreading of other toxins [132]. Given the prominence of the neurotoxic protein kallikrein-1 (KLK1) [178,179] in eulipotyphlan venoms, it is likely the main culprit behind symptoms like drop in blood pressure and subsequent paralysis [170], and as KLK1-related peptidases were also found in the saliva of *S. araneus* [168], it is likely that KLK-1 paralogs are present in the venoms of other European Eulipotyphla.

#### 3.4.3. Clinical Management

In the rare event of a bite, the area must be cleaned with plain water. Antibiotic therapy should be prescribed only in case of proven bacterial superinfection. Immunization for tetanus should always be evaluated and administered as needed.

### 3.5. Alien Species

The introduction of non-native venomous animal species can represent significant environmental and human health problems [180,181]. In fact, not only they can become invasive, potentially competing with native species and even leading to their extinction, but their venoms can represent a threat that physicians and PCCs are most likely unprepared to face. 

In this scenario, and exclusively considering vertebrates, alien fish species are currently the non-native species raising the most medical concerns. In this regard, the most important genus is likely *Pterois* (lionfish, originating from Indian and Pacific Ocean), which with its venomous sting can cause medical problems comparable to those induced by scorpionfish envenomation [182,183], thus requiring the same medical treatment. Catfish of the genus *Amerius*, a venomous fish originating from North America, should be considered a potential medical threat because, although non-deadly, it is able to inflict very painful stings and cause smooth muscle contraction [184].

## 4. Discussion

While several European vertebrates are able to cause potentially lethal envenomation/poisoning accidents, fatal outcomes can realistically be avoided with the rapid application of proper treatment. In fact, the majority of the envenomations caused by fish, amphibians and mammals generally lead to local symptoms that can be treated topically, and can resolve without sequelae if promptly treated. Regarding envenomations caused by snakes, the most likely to cause highly medically relevant systemic symptoms among the vertebrate groups considered here, rapid, appropriate intervention appears to be even more critical. However, emergency support services are often inefficient due to the lack of specific guidelines, and physicians are usually not properly trained in the management of animal-caused accidents. Additionally, clinical manifestations caused by animal toxins cover a very wide range of both local and systemic symptoms, and their interpretation is not trivial.

Rapidly recognizing these manifestations and knowing their management is of an increasingly important role in daily clinical practice, especially in medical centers located in areas where human–wildlife encounters are more likely (e.g., mountain, countryside, seaside), because it could make the difference in predicting the patient’s outcome. 

With this overview of the vertebrates most commonly involved in envenomation/poisoning events in Europe, the symptoms they can cause and their management, we hope to have provided a useful tool for the fast identification of the related clinical manifestations and the selection and application of the best approach to treat them.

## Data Availability

No new data were created or analyzed in this study. Data sharing is not applicable to this article.

## Supplementary Materials

The following supporting information can be downloaded at: https://www.mdpi.com/article/10.3390/life13061228/s1, Table S1: Information on European venomous and poisonous vertebrates of major medical relevance.
