# Supplementary material for: Venomous Bites, Stings and Poisoning by European Vertebrates as an Overlooked and Emerging Medical Problem: Recognition, Clinical Aspects and Therapeutic Management
Source: Life (Basel). 2023 May 23;13(6):1228. doi: 10.3390/life13061228 (PMC10305571; doi:10.3390/life13061228)
Supplement: Supplementary file 1 [file life-13-01228-s001.zip › life-2342435-supplementary.pdf]

Table S1. Information on European venomous and poisonous vertebrates of major medical relevance. For each taxon, details on distribution, symptoms they can cause, reported fatalities, and treatment are presented.

Common name	Genera	European distribution	Cutaneous reactions	Systemic reactions	Local therapy	Systemic therapy	Fatalities reported	Antivenom availability	Antivenom Name
Vipers	*Vipera*<br><br>*Montivipera*<br><br>*Macrovipera*	*Vipera*:<br>from Portugal to Russia, including Mediterranean islands (i.e., Sicily, Elba, Montecristo) and the UK.<br><br>*Montivipera*:<br>Milos archipelago (Greece) and Cyprus.<br><br>*Macrovipera*:<br>northeastern Greece and some Aegean islands.	Usually two fang marks, sometimes only one sign of a fang.<br><br>Pain, erythema, ecchymosis, ulcerations, edema, rarely necrosis and/or local infections.<br><br>Sometimes dry bites (bites without delivery of venom).	Anaphylaxis/anaphylactoid reaction, hypotension, hypertension, vomiting, diarrhea, neurotoxic signs, anxiety, hemorrhage, thrombosis, renal failure, myotoxicity, arrhythmias, ischemia, hyperglycemia. Rarely systemic infection.	Bandage in the field, Clean with hydrogen peroxide or with plain water. Avoid disinfection with alcohol or other chemicals substances. Check for any sign of compartmental syndrome. Antibiotics for any secondary super infection	In the absence of symptoms, only monitoring vital parameters (saturation, blood pressure, blood tests and ECG). Systemic therapies will be aimed at the type of internal damage. Antivenom is recommended from the G2 stage onwards.<br><br>Antitetanus check.	Yes	Yes*	Viper Venom Antitoxin<br><br>European viper venom antiserum<br><br>ViperaTAb<br><br>Viekvin<br><br>Viperfav<br><br>Snake Venom Antiserum<br><br>Vetel polysera<br><br>(for details, please refer to [22])
Colubrid snakes	*Malpolon*	Balkans, Greece, Portugal, Spain, southern France, Mediterranean north-western Italy and some Mediterranean islands.	Not double fang marks, but a semicircular/arceiform sign of a bite.<br><br>Erythema, edema associated with pain. Possible secondary super infection.	Usually neurotoxic symptoms, with oculomotor paralysis.	Clean with hydrogen peroxide or with plain water. Avoid disinfection with alcohol or other chemicals substances. Antibiotics for any secondary super infection	Symptomatic treatments and the treatments must be performed out according to the type of organ damage.	No	No	NA

Weever fish	*Trachinus* *Echiichtys*	Mediterranean Sea.	Usually poison entry point. Violent (stinging and stabbing) pain. Erythema, oedema, vescicles, ulceration and necrosis	Vaso-vagal reaction induced by the violent pain. Fever, arthralgia, cardiac arrhythmias and tonic-clonic seizures can also arise.	Put the affected part in hot water (maximum 40 °C), as the protein poison is thermo labile. Antibiotic treatment is justified only in case of super infections. Clean the part and remove any residual dirt.	The patient should reach the shore as soon as possible. The treatments are symptomatic. Check tetanus immunization. Antibiotics if infection.	Yes	No	NA
Stargazer fish	*Uranoscopus*	Mediterranean Sea.	Usually poison entry point. Pain and erythema .	Vaso-vagal reaction, and cardiac arrhythmias.	Hot water (maximum 40 °C) as the protein poison is thermolabile. Antibiotic treatment is justified only in case of super infections. Cleaning with plain water.	Reach the shore as soon as possible. Symptomatic treatment. Check tetanus immunization and antibiotics only in case of infection.	No	No	NA
Spiny dogfish	*Squalus*	Mediterranean Sea.	Erythema, oedema, pain, ulceration	Nausea, vomiting, cold sweats, muscular weakness	Reach the shore quickly. Clean the part with plain water and remove any residual dirt.	Out of the water as soon as possible. Symptomatic treatment. Tetanus immunization if needed.	Yes	No	NA
Scorpionfish	Scorpaenidae family	Mediterranean Sea.	The affected area becomes cyanotic, erythematous and edematous. Ulceration.	Cardio-circulator arrest, paralysis, hypotension, bradycardia, arrhythmias.	Clean the affected site with plain water. Use emetin hydrochloride, potassium permanganate and/or red congo to reduce the pain in the affected area	Hot water (maximum 40 °) to neutralize the toxins. Important monitoring cardiological activity injection of electrolytes. The treatment is symptomatic. Tetanus immunization should be provided if needed, as well as antibiotic therapy.	Yes	Yes	Equine Fab stonefish antivenom[¥]
Stingrays	*Dasyatis*	Mediterranean Sea.	Pain, erythema, edema. Possible bone involvement depending on the size of the barb.	Possible bone involvement, the symptoms vary according to the region of the sing and to the involved anatomic area.	Clean the wound with plain films, stitches if necessary.	Hot water to ne Systemic therapies will be aimed at the type of internal damage. Tetanus immunization if needed. Antibiotic prophylaxis is	Yes	No	NA

						advised.			
Toads, frogs and salamanders	*Salamandra*<br><br>*Bufo/Bufotes*<br><br>*Bombina*	*Salamandra*:<br>most of the continent, except the British Isles, much of North and East Europe and the major Mediterranean isles.<br><br>*Bufo/Bufotes*:<br>almost all of the continent, except Ireland and parts of Scandinavia.<br><br>*Bombina*:<br>large parts of Central, East and South-East Europe.<br><br>*Pleurodeles*:<br>Iberian Peninsula, except the northern mountain areas.	Signs and symptoms of irritative contact dermatitis	Possible burning of the mucous membranes of the oral cavity and of the nasal mucosa if inhalation of toxins and conjunctivitis if the eyes are touched.	Clean the part with fresh water. Hydrocortisone or Clobetasole to improve the local symptoms.	Usually only topical symptoms	No	No	NA
Shrews	*Sorex*<br><br>*Neomys*	All around Europe.	Pain, erythema, secondary infections.	Not reported.	Use iodopovidone to clean the bitten area and topical antibiotics as prophylactic therapy.	Tetanus immunization status should always be evaluated and immunization should be provided as needed.	No	No	NA

Viper Venom Antitoxin= developed against *Vipera berus*; European viper venom antiserum = developed against *Vipera ammodytes, Vipera aspis, Vipera berus, Vipera ursini, Macrovipera lebetina*; Vipera Tab= developed against *Vipera berus Vipera aspis, Vipera ammodytes, Vipera latastei*; Viekvin= developed against *Vipera ammodytes* and *Vipera berus*; Viperfav= developed against *Vipera berus, Vipera aspis, Vipera ammodytes*; Snake Venom Antiserum= developed against *Vipera ammodytes*; Vetel polysera= developed against *Vipera ammodytes*, *Macrovipera lebetina*, *Montivipera xanthina*

* except for *Macrovipera shweizeri.*

** The Scorpaenidae (scorpionfish) are a family of mostly marine fish that includes many of the world's most venomous species.

¥ In severe cases of scorpionfish and stonefish envenomation, equine Fab stonefish antivenom administered intramuscularly or intravenously is capable of neutralizing Indo-Pacific and Atlantic Scorpaenidae species.

NA = not available
